# Factors Influencing Cyanoacrylate Tissue Adhesive Outcomes for Corneal Thinning and Perforation

**DOI:** 10.3390/medicina61030492

**Published:** 2025-03-12

**Authors:** Anjali Om, Anjali Badami, Yuqing Wang, Xiangqin Cui, Soroosh Behshad, Joung Kim, Praneetha Thulasi

**Affiliations:** 1 Department of Surgery, Emory University, Atlanta, GA 30322, USA; 2 Washington Eye Specialists, Washington, DC 20017, USA; 3 Graduate Group in Epidemiology, University of California, Davis, CA 95616, USA; 4 Department of Biostatistics and Bioinformatics, Rollins School of Public Health, Emory University, Atlanta, GA 30329, USA; 5 Gavin Herbert Eye Institute, Department of Ophthalmology, University of California, Irvine, CA 92617, USA; 6 Department of Ophthalmology, Emory Eye Center, Emory University, Atlanta, GA 30322, USA; 7 Department of Ophthalmology and Visual Sciences, Washington University in St. Louis, St. Louis, MO 63141, USA

**Keywords:** corneal perforation, thinning, glue, cyanoacrylate glue

## Abstract

*Background and Objectives*: To report the outcomes of cyanoacrylate tissue adhesive (CTA) in patients with corneal perforations and thinning. *Materials and Methods*: A retrospective interventional study of 83 eyes treated with CTA for corneal thinning or perforation at a single institution between 2010 and 2020. Primary endpoints leading to CTA failure, visual acuity, and surgical outcomes were evaluated. *Results*: At presentation, 55 (66%) had frank perforations and 28 (34%) had thinning or desmetocele. Univariate analysis showed that only multiple CTA applications were associated with CTA failure (*p* = 0.047). Multivariate analysis did not show any statistically significant variables associated with CTA failure. No variables were associated with the need for future surgery at 30 days or any further point. Older patients (*p* = 0.005), use of topical steroids before gluing (*p* = 0.03), corneal thinning (vs. perforation) (*p* = 0.02), location of pathology (*p* = 0.048), and multiple CTA applications (*p* = 0.046) were associated with worse visual outcomes in univariate analysis. Multivariate logistic regression analysis showed that older age (OR 1.05) and use of topical steroids before gluing (OR 3.84) showed higher odds, and systemic immunosuppression (OR 0.08) and single (versus multiple) CTA application (OR 0.11) showed lower odds of worse visual acuity (BCVA ≥ 20/200). The presence of an anterior chamber prior to gluing was associated with spontaneous dislocation of CTA (*p* = 0.015). Spontaneous dislocation versus manual removal of glue was not associated with final visual acuity (*p* = 0.7), nor was duration of glue on the cornea (*p* = 0.2). *Conclusions*: CTA remains a mainstay of management in patients with corneal thinning or perforation. Only multiple CTA applications were associated with CTA failure, and duration of glue on the cornea was not associated with final visual acuity.

## 1. Introduction

Corneal thinning and perforation can result from severe insult to the cornea, such as infections, immune-mediated conditions, or trauma. Treating these patients is challenging since both the thinning and the underlying mechanism of the thinning need to be addressed concurrently. Cyanoacrylate tissue adhesive (CTA) is an excellent option to treat areas of thinning or perforations and has become a mainstay since its first published use in 1968 [1]. CTA quickly polymerizes upon contact with moisture, is readily available, and can be performed in a clinic setting, making it an ideal and often successful intervention in these complex patients [1,2,3]. While it is not Food and Drug Administration (FDA)-approved for use on the cornea, it is used ubiquitously. It is believed to act as a scaffold for fibroblasts to restore corneal integrity and may even have inherent bacteriostatic effects [4,5].

While CTA is a routine part of a cornea specialist’s armamentarium, systematic long-term analysis, especially addressing the natural course of CTA, is still being explored [6,7,8,9,10,11,12,13]. In this paper, we share our experience with CTA, focusing on presenting factors associated with treatment failure with CTA, as well as the natural course and visual outcomes of CTA. Our primary objective was to identify presenting factors that led to CTA failure. Our secondary objective was to describe visual acuity outcomes, the need for further surgical procedures, and the natural history of CTA after application.

## 2. Materials and Methods

This is a retrospective chart review of patients who underwent CTA in the clinic setting at a tertiary care center between 2010 and 2020. All patients with corneal thinning or perforation who underwent corneal gluing in a clinic setting were included. We excluded any patient glued in the emergency room or the hospital setting. All data were obtained and analyzed with Institutional Review Board approval. Patients were identified using Current Procedural Terminology (CPT) and diagnosis codes.

Demographic and presenting information, risk factors, and course of disease were documented. All patients were glued using n-Butyl-2-Cyanoacrylatemonomer (Histoacryl^®^, BBraun, Melsungen, Germany) and the glue was applied per the treating physician’s preference [2]. Glue was reapplied for a persistent leak, as judged by the treating physician. Subsequent surgical or medical interventions were decided by the treating physician. Visual acuity was assessed using a Snellen chart, and for patients who were unable to identify letters on a Snellen chart, visual acuity was assessed by their ability to count fingers, perceive hand motion, or see a light source [14]. A value of 20/200 or worse was used as a threshold for worse visual acuity since 20/200 is often used as a threshold for legal blindness.

For this study, we defined CTA failure as a persistent leak despite cornea gluing necessitating a keratoplasty. Subgroup analysis was performed for the definition of failure as patients who required a keratoplasty within 30 days of gluing, irrespective of the reason for surgery. Final visual acuity and glue outcomes were also examined.

For statistical analysis, visual acuity was converted to logarithm of the minimum angle of resolution (logMAR) [15,16]. Light perception and no light perception were not converted. All data analyses were conducted using R Studio software version 4.2.1.

Continuous variables were summarized as median (interquartile range, IQR), and categorical variables were summarized as counts (row percentages). Relevant statistical analysis (including Wilcoxon rank sum test to compare independent samples, Pearson’s chi-squared test, and Fisher’s exact test for hypotheses testing) were conducted for the following variables: age, sex, self-identified race, use of topical steroid, presence of desmetocele versus perforation, location, anterior chamber, etiology, and total number of applications per eye. Multivariate logistical regression analysis was performed based on the clinical relevance of a variable and statistical significance of the univariate analyses using these variables -age, sex, use of systemic immunosuppression, presence of desmetocele versus perforation, location, etiology, and total number of applications per eye. All results were regarded as statistically significant with a two-sided *p* value < 0.05.

## 3. Results

In total, 120 patients were identified of whom 83 patients met inclusion criteria. Demographic characteristics are presented in Table 1 and presenting corneal findings are described in Table 2. The median age of presentation was 65.5 years, with 42 (51%) female. A total of 53 (64%) self-identified as Caucasian, and 18 (22%) identified as African American. Of the total, 23% (21 patients) of patients were on systemic immunosuppression at presentation, and of these, 4 patients were undergoing chemotherapy for active cancer. A total of 42 patients (51%) were on topical corticosteroids and 2 patients (2.4%) were on topical NSAIDs at baseline.

At presentation, most patients had frank perforations (55, 66%). Most were central in location (43, 52%), and had either shallow or flat chambers (54, 65.1%). The primary etiology for corneal thinning or perforation was infectious (29, 35%), inflammatory (16, 19.3%), neurotrophic (31, 37.3%), and traumatic (7, 8.4%). Most patients had a single glue application (54, 65%) but 29% had CTA placed twice, and 6% had CTA placed three or more times.

A total of 47 eyes (56.6%) had keratoplasty in the follow-up period—43 had penetrating keratoplasty, and 4 had a patch graft. Sixteen (19.3%) patients had keratoplasty for a persistent leak despite CTA. Seven (8.4%) additional patients had a keratoplasty within one month of gluing for worsening of the underlying disease. The remainder of the patients (24 patients, 28.9%) had a keratoplasty to improve visual outcomes.

When CTA failure was defined as requiring surgery for persistent leak despite CTA, more than one CTA application was the only statistically significant variable (*p* = 0.047) in univariate analysis (Table 3). Multivariate analysis did not reveal any statistically significant variables. The multivariate analysis is listed in Table 4.

When evaluating for worse visual outcomes (vision equal to or worse than 20/200), older patients (*p* = 0.005), use of topical steroids prior to gluing (*p* = 0.03), corneal thinning (versus perforation) at presentation (*p* = 0.02), location of pathology (*p* = 0.048), and more than one CTA application (*p* = 0.046) were statistically significant in univariate analysis. These are described in Table 5. We also performed a multivariate logistic regression analysis for worse visual outcomes. Older age (OR 1.05) and use of topical steroids prior to gluing (OR 3.84) showed higher odds, and systemic immunosuppression (OR 0.08) and single (versus multiple) CTA application (OR 0.11) showed lower odds of worse visual acuity (BCVA ≥ 20/200). These variables are described in Table 6.

There were no statistically significant factors associated with CTA failure requiring surgery within 30 days of glue (either for a leak or for another etiology) or any future surgery.

The natural course of CTA was evaluated. CTA was more likely to fall off spontaneously in patients who had some anterior chamber prior to CTA placement (*p* = 0.015). The median time to glue falling off spontaneously (*n* = 49 patients) was 56.5 days (SD 95 days, range 6–360 days). Whether glue was removed in the operating room or allowed to fall off spontaneously was not associated with final visual acuity (*p* = 0.7). Similarly, the duration of glue was also not associated with final visual acuity (*p* = 0.2).

## 4. Discussion

In conclusion, our retrospective analysis of 83 patients undergoing CTA treatment for corneal thinning or perforation provides valuable insights into the factors associated with treatment outcomes. CTA emerged as a versatile and accessible intervention, effectively addressing corneal thinning or perforation in a clinic setting. However, significant gaps in our understanding of CTA management persist. Various approaches to managing the cornea following initial CTA application(s) require exploration—there is no consensus, for example, on whether to remove the glue at the slit lamp after the resolution of the acute process or wait for spontaneous dislocation. The impact of CTA on final visual acuity remains unknown. These questions may be answered if CTA is systematically evaluated in a large, relatively homogeneous population. Such a population is difficult to find within the context of CTA application.

Our primary objective was to identify presenting factors linked to CTA failure, defined as persistent leakage necessitating keratoplasty. Our study showed a failure rate of 19.2% (16 patients). Univariate analysis revealed a significant association between multiple CTA applications and treatment failure, underscoring the importance of identifying patients requiring alternative interventions early in the management process. Unlike other studies, we did not find associations between CTA failure and variables such as etiology, pathology location, or immunosuppressive use [7]. Multivariate analysis for CTA failure did not reveal statistically significant variables.

Our secondary objective focused on describing visual acuity outcomes, need for further surgical procedures, and the natural history of CTA after application. We evaluated various factors affecting poor visual acuity and found that advanced age, the use of topical steroids before gluing, corneal thinning (vs. perforation) at presentation, the location of pathology, and multiple CTA applications were identified as factors associated with worse visual outcomes (BCVA ≥ 20/200) in univariate analysis. However, multivariate analysis failed to show significance for the location of pathology, which indicates that the location of pathology did not have an independent contribution to worse visual outcomes. These findings are mostly consistent with clinical experience too—more severe findings requiring multiple gluing would be expected to have worse visual acuity. Similarly, older patients and those with local immunosuppression would also be expected to heal poorly.

One surprising finding in our study was that patients with corneal thinning at presentation had poorer visual acuity than those with frank perforation (*p* = 0.02). We hypothesize that the healing response in patients with residual stromal tissue may be different than in patients with frank perforations. We need further clinical and pathologic analysis using a larger cohort to tease out differences in this response.

We also evaluated the need for further surgical procedures. Our univariate analysis suggests, not surprisingly, that patients requiring multiple CTAs to stabilize a leak were more likely to require surgical intervention for persistent leak. Our clinical experience suggests that these patients often have a severe underlying disease, with a larger perforation or brisker leak. Our analysis, however, did not show statistically significant differences in the need for further surgery, whether within 30 days or at any future date.

Our analysis also highlighted that CTA was more likely to fall off spontaneously in patients with some anterior chamber prior to application. Exploring the natural course of CTA, we found that the duration of glue and the method of removal (spontaneous vs. operative) did not significantly impact final visual acuity, nor were any noted complications such as corneal neovascularization or worse stromal haze. We did not find a difference in visual acuities between spontaneous and manual glue removal; this suggests to us that the most important factor in determining the final visual acuity is the underlying insult, not the glue. Similarly, CTA duration was not associated with final visual acuity. The median time for spontaneous CTA detachment was 56.5 days.

Recent studies demonstrated varied success rates and complications associated with CTA. Yin et al. noted a success rate (as defined as an intact globe without surgical intervention) of 61% at 30 days in 140 eyes after glue application. They also noted that perforation (vs. thinning), large size of defect, and single application (vs. multiple) were associated with CTA failure [7]. Weiss et al. reported on 80 patients and noted a success of 44%, although they reported complications in 11% of the study group (elevated IOP or corneal infiltrates) [17]. Anchouche et al. presented a multicenter cohort study of 52 patients undergoing CTA and showed a success rate of 22% (as defined as tectonic stability of the globe without subsequent keratoplasty) [8]. Sadiq et al. reported 25 eyes with perforations; 56.5% were sealed with single gluing and 86.9% were sealed with repeat gluing [13]. Shankar et al. presented 24 patients with desmetocele (primarily non-perforated, 79.2%) and CTA was used successfully in 37.5% of eyes [18].

As evident from the above, the evaluation of all-comers presents a wide range of success rates (61–22%). Some of this variation can be attributed to different definitions of success, but a substantial part is also due to the diversity in underlying etiologies. A literature review suggests widely varying outcomes for different etiologies. For instance, Moorthy et al. reported a success rate of 37% in patients with corneal perforation secondary to herpetic keratitis [10]. In contrast, Singh et al. showed a success rate of 65% in patients with primarily bacterial keratitis [12]. Garg et al. successfully managed fungal keratitis with CTA in 63.6% of patients without further intervention [19]. Sharma et al., focusing on microbial keratitis in SJS, reported a success rate of only 20.7% with CTA [20]. In patients with rheumatoid arthritis-related perforations, Timlin et al. reported that 52% of patients required gluing, and while short-term outcomes were not presented, 60% of patients required a keratoplasty at 1 year [21]. These widely varying results underscore that conclusions drawn from this study may not be universally applicable to all patients requiring CTA, and outcomes may be better evaluated within the context of the underlying etiology.

The anatomy of corneal perforation may also be an unknown factor in the success of CTA. AlMaazmi et al. used anterior segment OCT (AS-OCT) to examine the anatomy of patients with seidel positive corneal perforations and formed versus flat anterior chambers (ACs) and noted that patients with a formed AC had an indirect communication between the AC and the exterior, as suggested by lamellar separation of the stroma, intrastromal pockets of glue, and epithelial bullae [22]. This group may have an entirely different outcome with CTA than a group with direct communication. While our study did not find a statistical association between the presence of a formed anterior chamber and CTA failure, the association with spontaneous dislocation after resolution suggests potential differences in the healing response.

Our study did not explore the relationship between the size of the corneal perforation and final visual and anatomic outcomes. We also did not explore whether the technique of gluing made a difference. Moreover, we did not have a large enough sample of patients with anterior segment OCTs at presentation to appreciate any anatomic differences in various presentations. The sample size and the variable etiologies for the initial corneal insult are limitations that we hope to address in a larger study in the future.

## 5. Conclusions

In summary, our study contributes to our understanding of CTA outcomes, offering guidance on patient selection and prognostic factors. While CTA remains a valuable intervention, caution is advised in cases requiring multiple applications, as these patients may benefit from alternative therapeutic approaches. Further research and long-term follow-up studies are warranted to refine treatment protocols and optimize outcomes for patients with corneal thinning or perforation.

## Figures and Tables

**Table 1:** Demographic characteristics of patients undergoing CTA for corneal thinning or perforation (*n* = 83).

Variable		*n* (%)
Median Age (IQR)		65.5 (50.89–75.67)
Female		42 (51%)
Self-identified Race		
	Caucasian	53 (64%)
	African American	18 (22%)
	Other	12 (14.5%)
Presence of Systemic Conditions		
	Thyroid disease	14 (13.5%)
	Diabetes	28 (26.9%)
	Active cancer undergoing therapy	4 (3.8%)
	Autoimmune disease	9 (8.7%)
Use of Systemic Immunosuppression		21 (23%)
	Corticosteroids	9 (10.8%)
	Non-corticosteroid	12 (14.6%)
Presence of Ocular Surface Disease		
	Dry eye disease	24 (29%)
	Neurotrophic keratopathy	31 (37.3%)
	Eyelid disorders	8 (9.6%)
	Prior corneal transplant	6 (7.2%)
	Limbal stem cell deficiency	4 (4.8%)
	Prior trauma	7 (8.4%)
Use of Ophthalmic Medications		
	Corticosteroids	42 (51%)
	Antimicrobials	41 (49.4%)
	Antifungals	2 (2%)
	Anti-amoebics	1(1%)
	Anti-glaucoma drops	20 (24.1%)
	Oral antivirals	6 (7.2%)
	Topical NSAIDs	2 (2.4%)

CTA = cyanoacrylate adhesive application; *n* = number; IQR = interquartile range; NSAIDs = non-steroidal anti-inflammatory drugs.

**Table 2:** Clinical characteristics in patients presenting with corneal perforation or thinning (*n* = 83).

Variable		*n* (%)
Presenting exam		
	Perforation	55 (66%)
	Thinning or Desmetocele	28 (34%)
Location of perforation/thinning		
	central	43 (52%)
	midperipheral	22 (27%)
	peripheral	18 (22%)
Anterior Chamber		
	Deep or shallow	63 (76%)
	Flat	15 (19%)
	Unknown	5 (6%)
Etiology		
	Infectious	29 (34.9%)
	Inflammatory	16 (19.3%)
	Neurotrophic	31 (37.3%)
	Traumatic	7 (8.4%)
Visual acuity at baseline (logMAR)		
	<1	29 (34.9%)
	≥1	54 (65.1%)
Number of applications per eye (median, range)		1 (1–3)
	one time	54 (65%)
	twice	24 (29%)
	thrice	5 (6.0%)
Duration of glue on cornea (days) (median, IQR)		45 (15.5, 91.0)

CTA = cyanoacrylate adhesive application; *n* = number; IQR = interquartile range.

**Table 3:** Univariate analysis of factors leading to CTA failure (defined as requiring surgery for persistent leak despite CTA) (*n* = 83).

Variable	Failure	Success	*p*-Value
Age	65.3 years	65.47 years	0.6
Female	11 (26%)	31 (74%)	0.11
African American race	6 (33%)	12 (67%)	0.10
Use of systemic immunosuppression			>0.9
No	12 (19%)	52 (81%)	
Yes	4 (21%)	15 (79%)	
Use of topical steroids			>0.9
No	8 (20%)	33 (80%)	
Yes	8 (19%)	34 (81%)	
Symptom			0.2
Perforation	13 (24%)	42 (76%)	
Thinning or desmetocele	3 (11%)	25 (89%)	
Location			0.7
Central	10 (23%)	33 (77%)	
Midperipheral	3 (14%)	19 (86%)	
Peripheral	3 (17%)	15 (83%)	
Anterior chamber			0.15
Absent	5 (33%)	10 (67%)	
Present	10 (16%)	53 (84%)	
Unable to assess	1 (20%)	4 (80%)	
Etiology			>0.9
Infectious	5 (17%)	24 (83%)	
Inflammatory	4 (25%)	12 (75%)	
Neurotrophic	6 (19%)	25 (81%)	
Traumatic	1 (14%)	6 (86%)	
Total number of CTA applications			0.047
More than once	9 (31%)	20 (69%)	
Once	7 (13%)	47 (87%)	

CTA = cyanoacrylate adhesive application; *n* = number.

**Table 4:** Multivariate regression analysis of factors leading to CTA failure (defined as requiring surgery for persistent leak despite CTA).

Variable	Adjusted OR	95% CI	*p* Value
African American race (vs. others)	0.34	0.08–1.42	0.1
Use of systemic immunosuppression	1.60	0.23–11.3	0.6
Use of topical steroids prior to glue	1.30	0.35–4.84	0.7
Perforation (vs. thinning)	0.61	0.12–2.96	0.5
Central location (versus peripheral)	0.87	0.14–5.29	0.9
Inflammatory (versus infectious)	0.80	0.09–6.85	0.8
One CTA application (vs. multiple)	3.16	0.83–12.00	0.1

CTA = cyanoacrylate tissue adhesive; OR = odds ratio; CI = confidence interval.

**Table 5:** Univariate analysis of factors affecting final visual acuity in patients undergoing CTA.

Variable	BCVA < 20/200 (*n* = 29)	BCVA ≥ 20/200 (*n* = 54)	*p*-Value
Age (median (IQR))	55.6 (46.7–65.5)	69.4 (57.5, 77.8)	0.005
Female	13 (31%)	29 (69%)	0.4
Race			0.7
African American	7 (39%)	11 (61%)	
Other races	22 (34%)	43 (66%)	
Use of systemic immunosuppression			0.2
No	20 (31%)	44 (69%)	
Yes	9 (47%)	10 (53%)	
Use of topical steroids at presentation			0.03
No	19 (46%)	22 (54%)	
Yes	10 (24%)	32 (76%)	
Anatomy at presentation			0.02
Perforation	24 (44%)	31 (56%)	
Thinning	5 (18%)	23 (82%)	
Location			0.048
Central	15 (35%)	28 (65%)	
Midperipheral	4 (18%)	18 (82%)	
Peripheral	10 (56%)	8 (44%)	
Anterior chamber			0.8
AC absent	6 (40%)	9 (60%)	
AC present	23 (37%)	40 (63%)	
Unknown	0	5	
Etiology			0.13
Infectious	8 (28%)	21 (72%)	
Inflammatory	7 (44%)	9 (56%)	
Neurotrophic	9 (29%)	22 (71%)	
Traumatic	5 (71%)	2 (29%)	
Number of CTA applications			0.046
More than once	6 (21%)	23 (79%)	
Once	23 (43%)	31 (57%)	

CTA = cyanoacrylate tissue adhesive; BCVA = best corrected visual acuity; *n* = number; IQR = interquartile range.

**Table 6:** Multivariate regression analysis of factors predicting poor final visual acuity (as defined as BCVA ≥ logMAR 1).

Variable	Adjusted OR	95% CI	*p* Value
Older age	1.05	1.01–1.10	0.01
African American race (vs. others)	2.52	0.56–11.32	0.2
Use of systemic immunosuppression	0.08	0.01–0.58	0.01
Use of topical steroids prior to glue	3.84	1.06–14.7	0.049
Perforation (vs. thinning or desmetocele)	0.33	0.08–1.46	0.14
Central location (versus peripheral)	2.59	0.52–12.98	0.2
Inflammatory (versus infectious)	1.13	0.15–8.59	0.9
One CTA application (vs. multiple)	0.11	0.02–0.56	0.008

CTA = cyanoacrylate tissue adhesive; BCVA = best corrected visual acuity; OR = odds ratio; CI = confidence interval.

## Data Availability

The data presented in this study are available on request from the corresponding author due to privacy reasons.
